# Evolution of Grain Boundary Character Distribution in B10 Alloy from Friction Stir Processing to Annealing Treatment

**DOI:** 10.3390/ma17051134

**Published:** 2024-02-29

**Authors:** Wen Feng, Junjie Zhou, Shihao Wang, Ting Sun, Tianyu Zhao, Yingying Jiang

**Affiliations:** 1 School of Mechanical Engineering, Jiangsu Ocean University, Lianyungang 222005, China; aeeeolia@outlook.com (J.Z.); 2023220516@jou.edu.cn (T.S.); 2023220531@jou.edu.cn (T.Z.); 2021121077@jou.edu.cn (Y.J.); 2 Jiangsu Institute of Marine Resources Development, Lianyungang 222005, China; 3 Makarov College of Marine Engineering, Jiangsu Ocean University, Lianyungang 222005, China; 2022221706@jou.edu.cn

**Keywords:** B10 alloy, friction stirring processing, grain boundary engineering, grain boundary character distribution, thermomechanical processing

## Abstract

In this study, the grain boundary character distribution (GBCD) of a B10 alloy was optimized, employing thermomechanical processing consisting of friction stirring processing (FSP) and annealing treatment. Using electron backscatter diffraction, the effects of rotational speed of FSP and annealing time on the evolution of GBCD were systematically investigated. The GBCD evolution was analyzed concerning various parameters, such as the fraction of low-Σ coincidence site lattice (CSL) boundaries, the average number of grains per twin-related domain (TRD), the length of longest chain (LLC), and the triple junction distribution. The experimental results revealed that the processing of a 1400 rpm rotational speed of FSP followed by annealing at 750 °C for 60 min resulted in the optimum grain boundary engineering (GBE) microstructure with the highest fraction of low-Σ CSL boundaries being 82.50% and a significantly fragmented random boundary network, as corroborated by the highest average number of grains per TRD (14.73) with the maximum LLC (2.14) as well as the highest J2/(1 − J3) value (12.76%). As the rotational speed of FSP increased from 600 rpm to 1400 rpm, the fraction of low-Σ CSL boundaries monotonously increased. The fraction of low-Σ CSL boundaries first increased and then decreased with an increase in annealing time. The key to achieving GBE lies in inhibiting the recrystallization phenomenon while stimulating abundant multiple twinning events through strain-induced boundary migration.

## 1. Introduction

Grain boundary engineering (GBE), as a widely used microstructure control approach, is derived from the idea of grain boundary control and design, initially introduced by Watanabe [1]. Over the past few decades, GBE has been successfully employed to alleviate the susceptibility of materials to a variety of intergranular-related degradation events, including intergranular corrosion [2,3,4,5], fatigue [6,7], precipitation [8,9,10], embrittlement [11,12], weld cracking [13], etc. The fundamental idea behind GBE is to increase the fraction of low-Σ coincidence site lattice (CSL) boundaries and break the connectivity of a random boundary network in face-centered cubic metallic materials with low-stacking fault energies through suitable thermomechanical processing (TMP) routes. It is to be noted here that although the structure and energy of grain boundary cannot be entirely determined via the Σ-value alone, these low-Σ CSL boundaries are frequently regarded as special. Recent research shows that only a portion of low-Σ CSL boundaries are exceptional [14,15]. However, much experimental data indicates that, compared to random boundaries (RBs), low-Σ CSL boundaries exhibit more excellent resistance against grain boundary degradation because they have a higher ordering degree, a lower interface energy, and a smaller free volume [16,17,18].

Annealing twin boundaries, also known as Σ3 boundaries, are the primary constituents of low-Σ CSL grain boundaries and play a critical role in achieving the GBE microstructure. Different TMP routes (pre-deformation followed by annealing treatment) have been performed to increase the fraction of Σ3 boundaries in the material by rearranging the grain boundary structure through mutual interaction between other existing twins or generating new annealing twins [19,20]. The growth and interaction of Σ3^n^ (n = 1, 2, 3) boundaries are involved in the former, also called multiple twinning. It is regulated by the relationship ΣA + ΣB → Σ(A/B) or ΣA + ΣB → Σ(A × B) when it comes to dissociating or merging low-Σ CSL grain boundaries. For example, the interaction between two Σ3 boundaries forms a Σ9 boundary because of geometric constraints at the triple junction. Depending on the following rule: Σ9 + Σ3 → Σ(9/3) or Σ3 + Σ9 → Σ(3 × 9), a new Σ3 boundary or a Σ27 boundary is formed when a Σ9 boundary interacts with another Σ3 boundary. In the latter case, Σ3 boundaries are formed and grown in a direction parallel to the direction of grain boundary migration. Σ3 boundaries formed under this condition often manifest as isolated features within the grains and cannot be incorporated into the random boundary network.

Strain recrystallization and strain annealing are the main TMP routes to optimize grain boundary character distribution (GBCD) [21]. The process of strain recrystallization consists of sequentially introducing a medium amount of deformation followed by a short duration of high-temperature annealing that results in the nucleation and growth of strain-free grains. In contrast, strain annealing involves a longer annealing time at a lower temperature to initiate the phenomenon known as strain-induced boundary migration (SIBM). Until now, uniaxial tension/compression and cold rolling are the pre-deformation techniques typically employed to introduce stored energy into the material to provide the driving force for optimizing GBCD during the annealing process [22,23,24]. Nevertheless, conventional TMP finds fabricating complex hollow components or customized parts with near-net shapes challenging.

Based on the fundamentals of friction stir welding, Mishra et al. [25] developed friction stir processing (FSP), a novel solid-state plastic deformation technique that is energy-efficient and environmentally friendly, to modify the microstructure of metallic materials. The FSP technique has several advantages. First, crack and porosity-free modified layers can be produced using FSP as it avoids the bulk melting of the material. Second, significant grain refinement in the processed zone can be achieved due to recovery and dynamic recrystallization during the FSP. Third, the depth and area of the processed zone can be flexibly controlled by varying the tool size, which sets it apart from other severe plastic deformation techniques like high-pressure torsion, accumulative roll bonding, and equal channel angular pressing. The whole specimen can be processed to produce a sizeable bulk material, or we can only process the material’s surface, and the shape of the material remains unchanged.

So far, FSP has been chiefly utilized for surface composite manufacturing and mechanical property enhancement of metallic materials [26,27,28]. To the authors’ knowledge, no comprehensive analysis of the GBCD optimization through FSP combined with annealing treatment has yet been carried out. A commercial B10 alloy was selected as a model alloy for this study because it is a face-centered cubic metal with low-stacking fault energy, in which Σ3 boundaries are easily formed. In other words, other face-centered cubic alloys with low- to medium-stacking fault energy (e.g., B8, B20, Cu-Sn alloy, and austenitic stainless steel, etc.) are also suitable as experimental materials for this study. Concerning this, the main objective of this study is to explore the application feasibility of the combination of FSP and annealing treatment to generate a GBE microstructure in B10 alloy. To achieve this goal, a series of TMP experiments with different rotational speeds of FSP and annealing time were carried out in B10 alloy, and the GBCD evolution was analyzed under various conditions.

## 2. Experimental Materials and Procedure

### 2.1. Materials and Processing

**Figure 1:**
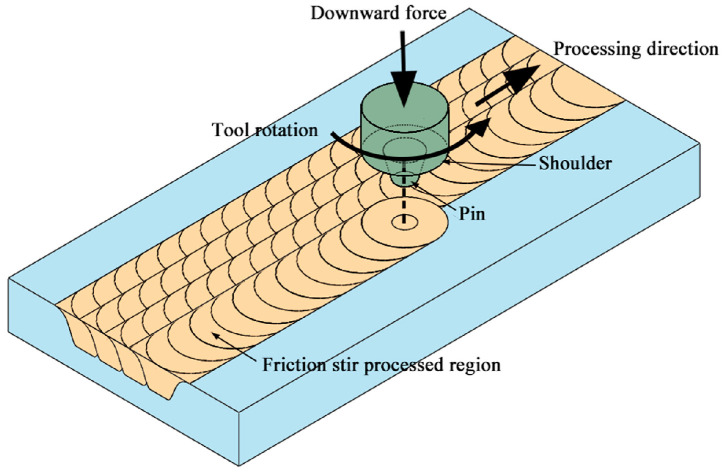
Schematic diagram of the friction stir processing.

Table 1 provides the chemical composition (in wt. %) of the commercial B10 alloy. The as-received material was fabricated into several blocks of 150 mm × 80 mm × 16 mm using wire-cut electrical discharge machining. To get rid of microstructural inhomogeneity and any potential residual strain, each block was cold rolled using a 30% thickness reduction and then annealed at 800 °C for 30 min, followed by an immediate quenching with water, which was subsequently designated as the base material (BM). Before FSP, the BM surface was ground using #1000 grade SiC abrasive to remove oxides and then cleaned with ethanol. The BM specimen was friction stir processed (FSPed) using a SCB LM2217-2D-12T FSW Machine (Sooncable Technology Group Co., Beijing, China). During the FSP procedure, a non-consumable rotating tool with a customized pin and shoulder was inserted into the B10 alloy, as shown in Figure 1. After frictional heat softened the B10 alloy, the tool moved in a predefined path to process the B10 alloy in the target area. An overlapping multi-pass FSP was carried out to enlarge the processed zone of B10 alloy, and the overlap rate between adjacent passes was about 50%. The rotating tool was made of a high-hardness, wear-resistant W-Re alloy to prevent an Fe element contamination of the processed zone. The shoulder diameter of the rotating tool was 14 mm. The top diameter, bottom diameter, and length of the tapered threaded pin were 4 mm, 6 mm, and 5 mm, respectively. The parameters of FSP are as follows: the tool plunge depth was 0.3 mm, the tool transverse speed was 150 mm/min, and the rotational speeds of the tool were varied from 600 rpm to 1400 rpm in steps of 200 rpm, which will be expressed for clarity in the format of S600, S800, S1000, S1200, and S1400, respectively. All FSPed specimens were subsequently annealed to modify the GBCD of the B10 alloy. Table 2 lists the detailed process parameters and specimen designation.

### 2.2. Microstructural Characterization

Microstructures of the specimens were characterized on the cross-section perpendicular to the processing direction. The grain boundary misorientation in the middle of the stir zone of the specimen was analyzed using an Oxford Nordlys electron backscattered diffraction (EBSD) accessory on a Carl Zeiss MERLIN Compact scanning electron microscope (SEM). For removing the surface strained layer, each specimen for the EBSD test was initially ground with abrasive papers up to #1000 grade, followed by mechanical polishing with 1 μm diamond paste, and finally electro-polished in a solution (17.5% ethanol and 82.5% phosphoric acid in volume) at the voltage of 2.1 for 10 min. Based on the grain size of the specimens, EBSD scans were performed with a step size of 0.3 μm or 2.0 μm. At least three scans in different regions were carried out on each specimen for statistical significance, and an average was utilized to depict the data presented in this study. HKL Channel 5 analysis software version 5.0.9.1 was employed for post-processing and the noise removal was less than 5% for each specimen. The grain boundaries were determined, considering a misorientation of greater than 5°. The grain size was assessed using circle equivalent grain diameter. The tolerance for low-Σ CSL boundaries was identified using the Brandon criterion [29]. Grain boundaries with 3 ≤ Σ ≤ 29 were considered low-Σ CSL boundaries. RBs were defined with the stipulation that grain boundary misorientation was greater than 15° and grain boundaries were not low-Σ CSL boundaries.

Twin-related domain (TRD) analysis with the parameters of average number of grains per TRD and length of longest chain (LLC) was applied to quantitatively evaluate the extent of multiple twinning using the ARPGE software version 1.7 [30]. An individual TRD was defined as all grains inside a given cluster interconnected through the Σ3^n^ (n = 1, 2, 3) boundaries. Furthermore, triple junction distribution has been carried out to provide insights into the connectivity of a random boundary network in the microstructure. According to Fortier [31], there are four types of triple junctions: J0 (0-CSL), J1 (1-CSL), J2 (2-CSL), and J3 (3-CSL). Here, a triple junction with n number of low-Σ CSL boundaries at the intersection is represented by n-CSL. Based on the fractions of J2 and J3-type triple junctions, the J2/(1 − J3) parameter was calculated for each specimen to assess random boundary network connectivity [32].

## 3. Results and Discussion

### 3.1. Microstructure of BM

**Figure 2:**
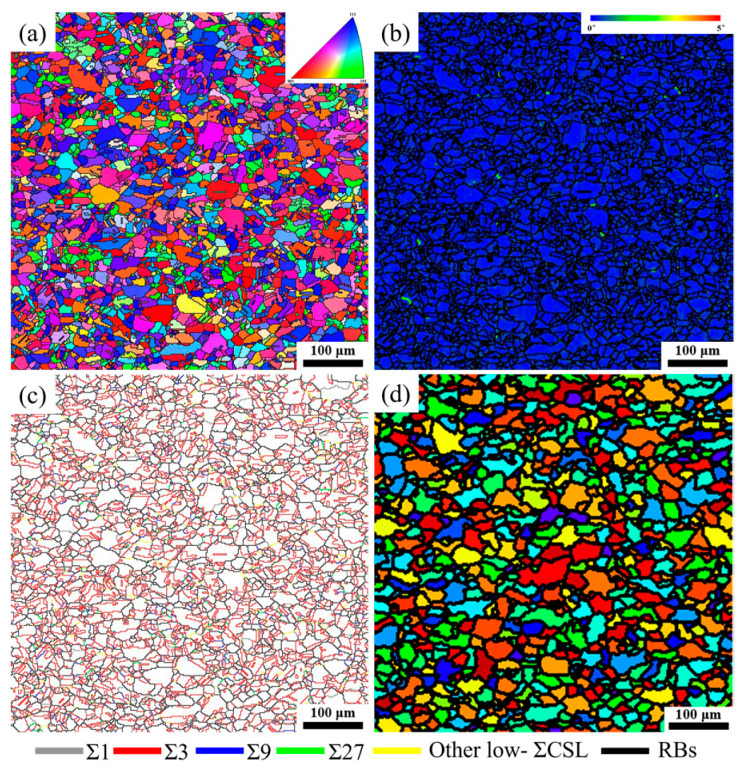
Microstructure of the BM specimen: (**a**) IPF map, (**b**) local misorientation map, (**c**) grain boundary reconstruction map, and (**d**) TRD map.

Figure 2 displays the microstructure of the BM specimen. It can be deduced from the inverse pole figure (IPF) map (Figure 2a) that the grains were oriented in random directions for the BM specimen. Furthermore, approximately equiaxed grains with parallel-side or straight twin boundaries were distributed almost homogeneously throughout the whole microstructure, and the grain size exhibited a unimodal distribution, with an average of 16.47 μm (excluding the annealing twins). The meager value of local misorientation, which indicates the absence of stored energy in the BM specimen, is displayed in the local misorientation map (see Figure 2b). Figure 2c presents the grain boundary reconstruction map of the BM specimen. Here, grey, red, blue, green, yellow, and black lines represent Σ1, Σ3, Σ9, Σ27, other low-Σ CSL boundaries and RBs, respectively. The grain boundary fraction in the BM specimen was statistically analyzed, showing that the fraction of low-Σ CSL boundaries was 52.82%, consisting of 44.70% Σ3 boundaries and 5.30% (Σ9 + Σ27) boundaries. The fraction of (Σ9 + Σ27) boundaries is much lower than that of Σ3 boundaries, but Σ9 and Σ27 boundaries generally serve as geometrical requirements for the regeneration of Σ3 boundaries. A low fraction of (Σ9 + Σ27) boundaries suggests that low-Σ CSL boundaries did not break the connectivity of the random boundary network in the BM specimen after recrystallization treatment. The TRD map of the BM specimen is displayed in Figure 2d. Detailed quantitative data reveals that the BM specimen exhibited a limited development of multiple twinning, as evidenced by the average number of grains per TRD and LLC of 2.26 and 1.07, respectively. The RBs prefer the formation and propagation of intergranular cracks compared to low-Σ CSL boundaries [33,34]. It suggests that the BM specimen was susceptible to intergranular failure. Thus, to enhance the resistance of the BM specimen to intergranular degradation, its GBCD needs to be further optimized.

### 3.2. Microstructural Analyses of FSPed B10 Alloy

**Figure 3:**
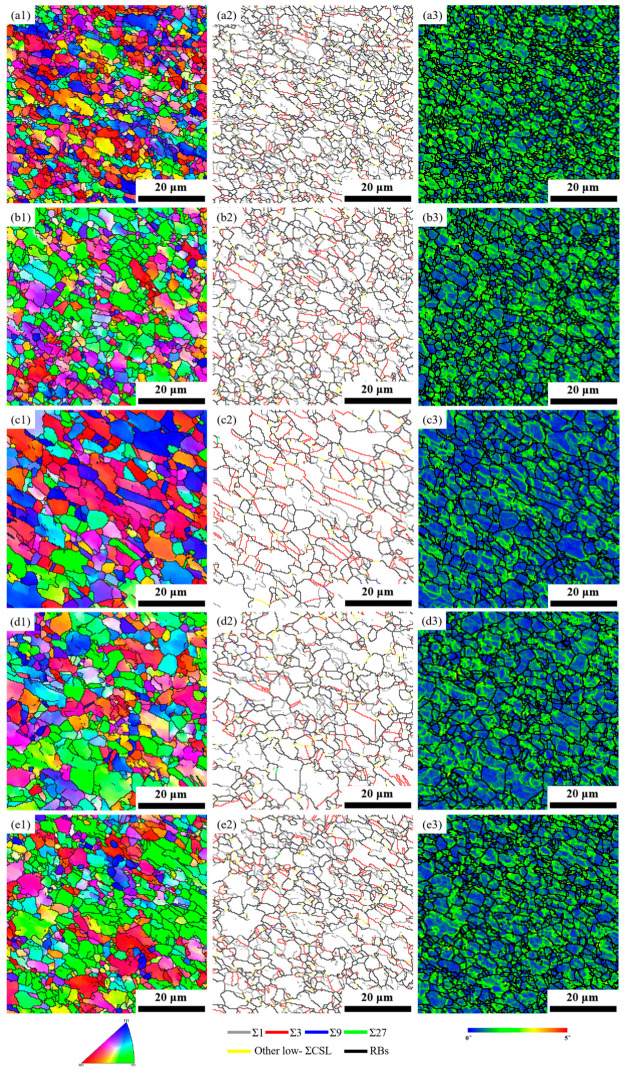
IPF, grain boundary reconstruction, and local misorientation maps for the FSPed specimens subjected to different rotational speeds: (**a1**–**a3**) S600, (**b1**–**b3**) S800, (**c1**–**c3**) S1000, (**d1**–**d3**) S1200, and (**e1**–**e3**) S1400.

Figure 3 shows the IPF, grain boundary reconstruction, and local misorientation maps of the FSPed specimens subjected to different rotational speeds. Detailed information about the grain size and grain boundary character of the FSPed specimens are given in Table 3. Compared to the BM specimen, the grains were significantly refined in the FSPed specimens, which is a result of the occurrence of dynamic recrystallization (DRX) by severe plastic deformation and high frictional heat during FSP (Figure 3(a1–e1)). Nonetheless, the microstructure was partially equiaxed due to an incomplete recrystallization process. Further detailed observation revealed that when the rotational speed increased, the grain size initially increased and then decreased (Table 3). It is well known that increasing rotational speed would increase both the strain rate and the heat input. Accelerating dynamic recrystallization at a higher strain rate would, on the one hand, promote grain refinement. Conversely, the increased heat input would encourage grain growth. The final grain size results from the two factors working against one another. Consequently, the main factor determines how the grain size in the FSPed specimen changes with rotational speed. The maximum grain size was obtained at a rotational speed of 1000 rpm, as indicated in Figure 3 and Table 3. Hence, an increase in heat input causes a more significant impact on the grain size than the increased strain rate when the rotational speed is between 600 and 1000 rpm. The effect of increasing strain rate overwhelms that of the increased heat input when the rotational speed is between 1000 and 1400 rpm, causing a decrease in grain size.

Figure 3(a2–e2) illustrates the grain boundary reconstruction maps of the FSPed specimens, and Table 3 summarizes the GBCD statistics. The fractions of low-Σ CSL boundaries in the FSPed specimens decreased significantly compared to the BM specimen (58.3%) and first increased and then decreased with the increase of rotational speed. Specifically, the fractions of low-Σ CSL boundaries in the specimens S600, S800, S1000, S1200, and S1400 were 12.57%, 19.02%, 29.51%, 22.21%, and 20.55%, respectively. In addition, the fraction of Σ1 and low-Σ CSL boundaries presented an opposite trend with increased rotational speed.

**Figure 4:**
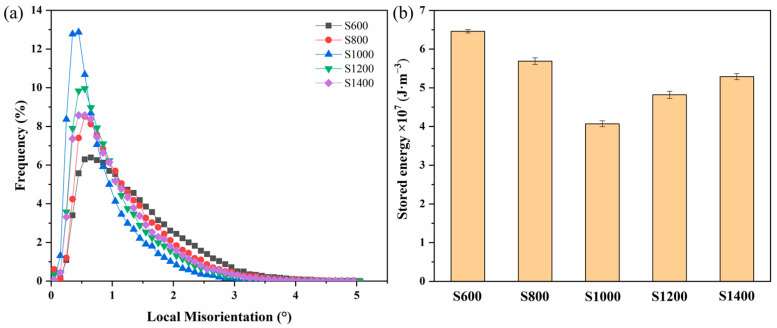
Local misorientation plot (**a**) and stored energy (**b**) of the FSPed specimens.

The local misorientation maps derived from the FSPed specimens with different rotational speeds are displayed in Figure 3(a3–e3). Using a rainbow color coding, the residual strain distribution can be qualitatively evaluated based on the magnitude of local misorientation. As seen in Figure 3(a3–e3), the local misorientations in all the FSPed specimens were heterogeneous, and the strains were mainly located around grain boundaries. The local misorientation data against the rotational speed are plotted in Figure 4a. A detailed analysis of Figure 4a can reveal that as rotational speed increased, the peak and width of the local misorientation distribution changed to a higher angle and were enlarged, respectively. The average value of local misorientation can be used to calculate the stored energy E of the microstructure with the formulation [35] expressed as

(1) $$E=\frac{\alpha \theta Gb}{2d}$$

where the grain boundary type determines the parameter α. Generally, the α values of tilt boundaries, twist boundaries, and the two types of mixed boundaries are 2, 4, and 3, respectively. θ is the average local misorientation; G represents the shear modulus; b means the burgers vector; d stands for the step size. According to the studies [36,37,38], the values of α, G, and b were 3, 39 GPa, and 0.128 nm, respectively. In this study, the step size d was 0.3 μm. Figure 4b shows the stored energy of the FSPed specimens as calculated by Equation (1). The stored energy in the FSPed specimens first decreased and then increased with the increasing rotational speed. The combined effects of dislocation–dislocation interactions, dynamic recovery, and dynamic recrystallization cause a change in the stored energy. For specimen S1000, the lowest stored energy value is attributed to the weak dislocation–dislocation interactions and the formation of numerous strain-free DRX grains.

### 3.3. Effect of Rotational Speed on the Evolution of GBCD

**Figure 5:**
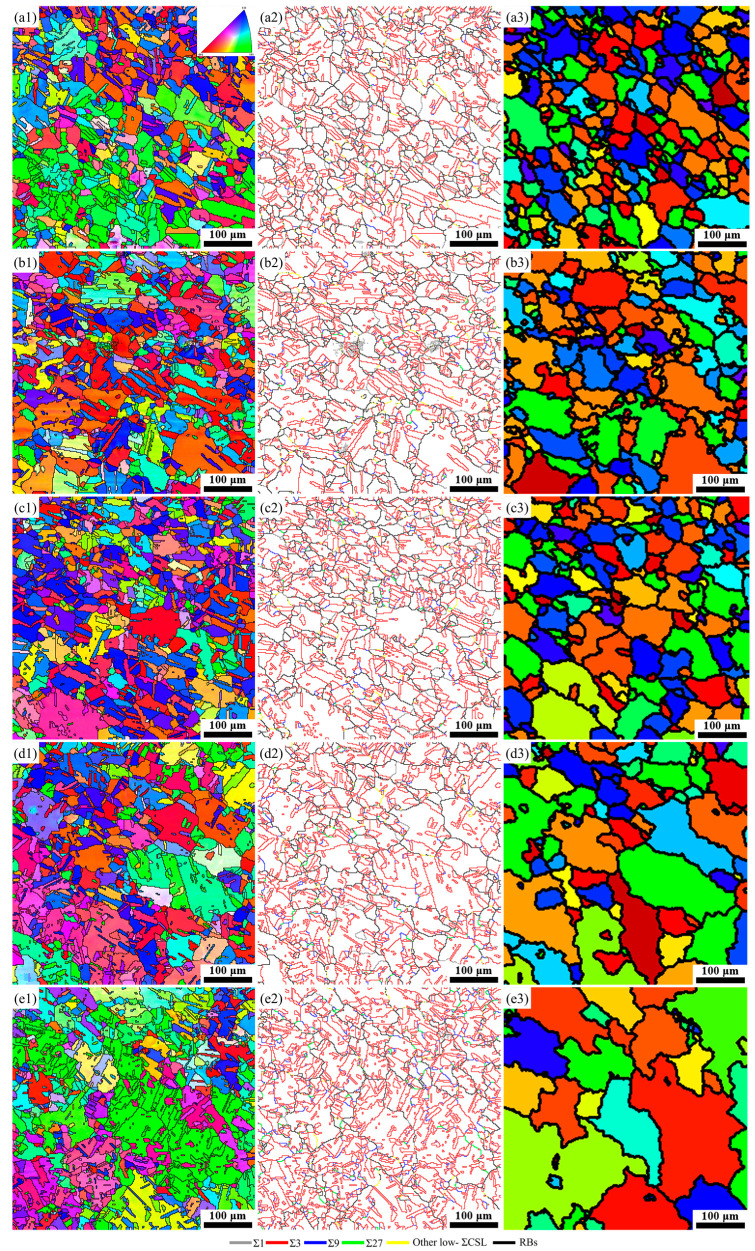
IPF, grain boundary reconstruction, and TRD maps of (**a1**–**a3**) A-1, (**b1**–**b3**) A-2, (**c1**–**c3**) A-3, (**d1**–**d3**) A-4, (**e1**–**e3**) A-5 specimens.

**Figure 6:**
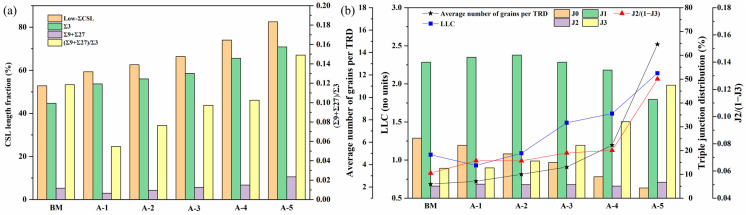
(**a**) The GBCD statistics, (**b**) average number of grains per TRD and LLC, and triple junction distribution of the TMP specimens with different rotational speeds. Data from the BM specimen are also shown for comparison.

Figure 5 presents the IPF, grain boundary reconstruction, and TRD maps for specimens that underwent various rotational speeds following annealing treatment at 750 °C for 60 min. The effect of rotational speed on the GBCD evolution after annealing treatment is depicted in Figure 6. The IPF maps in Figure 5(a1–e1) show that the texture of each specimen was nearly random and weak, suggesting that the formation of low-Σ CSL boundaries is independent of texture. The average grain size increased from 25.00 μm to 26.42 μm, 27.91 μm, 34.22 μm, and 34.74 μm when the rotational speed was 600 rpm, 800 rpm, 1000 rpm, 1200 rpm, and 1400 rpm. The results indicate that during annealing treatment, specimens A-1 and A-2 experienced extensive recrystallization, whereas grain growth occurred in specimens A-3, A-4, and A-5. This is because the stored energies in specimens S600 and S800 were higher than those in specimens S1000, S1200, and S1400 (see Figure 4b). Further observation reveals that specimens A-1, A-2, and A-3 have relatively uniform grain size distributions, while specimens A-4 and A-5 have bimodal distributions with local abnormal grain growth. For specimens A-1 and A-2, extensive recrystallization occurred during annealing treatment as the imparted stored energies were higher than the critical stored energy for recrystallization. Thus, the grain size distributions in specimens A-1 and A-2 presented unimodal distributions. The number of grains that grew rapidly via SIBM in specimen A-3 was far less due to the lowest stored energy, resulting in relatively uniform grain growth. In specimens A-4 and A-5, the number of grains that grow rapidly via SIBM significantly increased as the stored energies were further increased. Therefore, after annealing treatment, the rapidly growing grains develop into large grain clusters, forming a bimodal grain size distribution structure.

As shown in Figure 5(a2–c2), the majority of Σ3 boundaries in the form of straight or parallel-sided lines terminated within a grain for specimens A-1, A-2, and A-3. As a result, the microstructure of these specimens was characterized by a well-connected random boundary network. In contrast, the morphology of Σ3 boundaries in specimens A-4 and A-5 was convoluted and interconnected, and the Σ3^n^ (n = 1, 2, 3) boundaries were essential components of a random boundary network (Figure 5(d2,e2)). The fraction of low-Σ CSL boundaries, Σ3 boundaries, (Σ9 + Σ27) boundaries, and the ratio of (Σ9 + Σ27)/Σ3 increased when the rotational speed increased from 600 rpm to 1400 rpm. The highest fractions of low-Σ CSL boundaries, Σ3 boundaries, (Σ9 + Σ27) boundaries, and the largest (Σ9 + Σ27)/Σ3 value were obtained in the specimen A-5 (see Figure 6a).

Figure 5(a3–e3) presents the reconstructed TRD maps for the TMP specimens with different rotational speeds. The TRD sizes of specimens A-4 and A-5 were noticeably larger than those of other conditions. The average number of grains per TRD and LLC have been calculated from TRD maps of various specimen conditions for quantitative analysis, as displayed in Figure 6b. The average number of grains per TRD for specimen A-1 was 2.51, which is considerably less than the value for specimen A-5 (15.9). Similarly, specimen A-5 exhibited the largest LLC, indicating the highest order of GBE extent. As previously mentioned, a higher LLC value denotes a higher order of twinning for specimen A-5, resulting in a larger TRD size.

Triple junction distributions for the BM specimen and TMP specimens with various rational speeds are plotted in Figure 6b. It can be seen that, for all the specimen conditions, except specimen A-5, the majority of triple junctions were composed of type J0 and J1, suggesting a relatively unclustered spatial distribution of low-Σ CSL boundaries in the microstructure (see Figure 2c and Figure 5(a2–e2)). Specimen A-5 presented the highest fraction of J3-type triple junctions and the greatest J2/(1 − J3) value compared to the other specimens. This means that the microstructure exhibits a significant clustering of low-Σ CSL boundaries that polarizes toward the J3-type triple junction, and the connectivity of the random boundary network is substantially disrupted in the specimen A-5 (refer to Figure 5(e2)). Furthermore, it can be observed that, compared to J0, J1, and J3-type triple junctions, the fraction of J2-type triple junctions was low and comparatively constant under all specimen conditions. This can be interpreted in light of the geometrical constraints given by the addition rule, which states that the third boundary must be another low-Σ CSL boundary if two low-Σ CSL boundaries interact at a triple junction. The saturation of the J2-type triple junction and a sharp increase in the J2/(1 − J3) value would manifest a GBE microstructure.

Many researchers [39,40,41] have confirmed that the recovery process rather than the recrystallization process is conducive to optimizing the GBCD, which is determined by the level of stored energy throughout the grains in the microstructure. As stated before, extensive recrystallization occurred in specimens A-1 and A-2, while grain growth happened in specimens A-3, A-4, and A-5 during annealing. Therefore, specimens A-3, A-4, and A-5 are expected to have higher fractions of low-ΣCSL boundaries than specimens A1 and A2. It can be explained that during the recovery process, strain-induced boundary migration occurred and pre-existing Σ3^n^ (n = 1, 2, 3) boundaries interacted with each other, leading to multiple twinning events, e.g., Σ3 + Σ3 → Σ9, Σ3 + Σ9 → Σ3 or Σ3 + Σ9 → Σ27, which is the primary mechanism for increasing the fraction of low-ΣCSL boundaries and disrupting the random boundary network connectivity.

As the grain boundaries inside the TRD are connected by the Σ3^n^ (n = 1, 2, 3) boundaries, specimens A-4 and A-5 presented a higher average number of grains per TRD and LLC values than other specimens (Figure 6b). Nevertheless, specimen A-3 has a relatively low fraction of low-Σ CSL boundaries, considering the stored energy in specimen S1000 provides insufficient driving force for grain boundaries to migrate over long distances, which results in the extent of multiple twinning being much lower during annealing treatment. Multiple twinning can occur behind the migrating grain boundaries due to the existing TRDs in the microstructure growing during such an SIBM. A lower fraction of low-Σ CSL boundaries, formed via insufficient SIBM, leads to the insignificant disruption in the connectivity of a random boundary network as substantiated by a lower J2/(1 − J3) value. Conversely, for specimens A-1 and A-2, static recrystallization occurs during subsequent annealing treatment as stored energy exceeds the critical value, which results in the formation of new strain-free grains with RBs and the removal of pre-existing Σ3 boundaries. As new strain-free grains prefer normal grain growth, the distances for the migration of RBs are relatively limited, restricting the formation and interaction of low-Σ CSL boundaries. Therefore, the product microstructure in specimens A-1 and A-2 yields a lower fraction of low-Σ CSL boundaries, average number of grains per TRD and LLC values, with a concurrent increase in the connectivity of the random boundary network, as confirmed by its lower J2/(1 − J3) value. These results align with the data from previous research [42]. Hence, during GBE-type TMP, it is crucial to optimize the stored energy to instigate prolific SIBM and prevent recrystallization. Namely, the imparted stored energy should be less than the critical value required for recrystallization nucleation.

### 3.4. Effect of Annealing Time on the Evolution of GBCD

**Figure 7:**
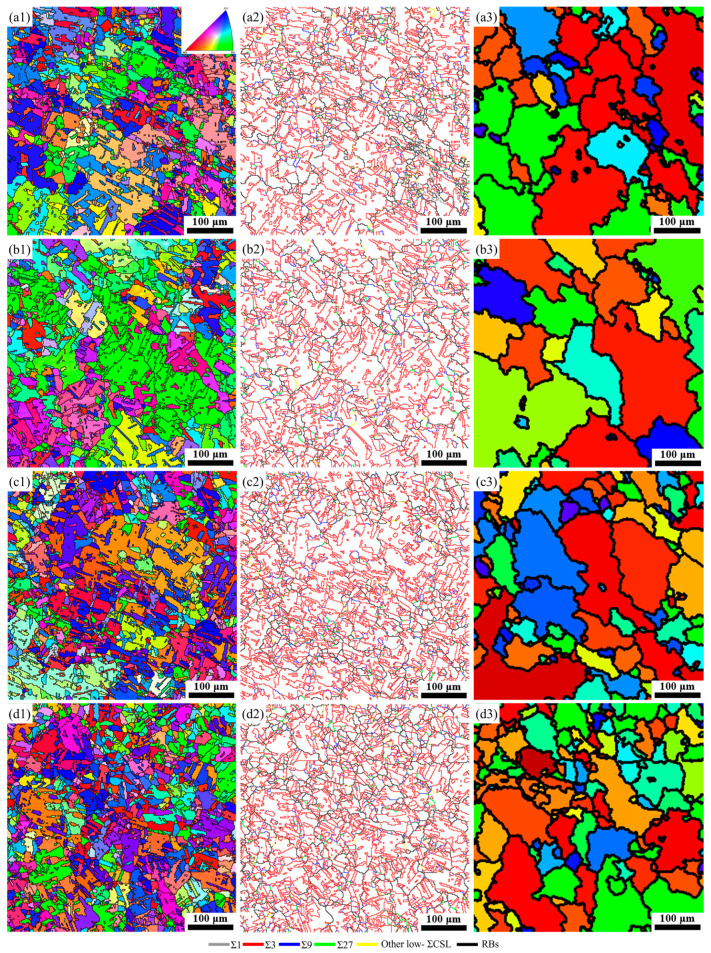
IPF, grain boundary reconstruction, and TRD maps of (**a1**–**a3**) B-1, (**b1**–**b3**) B-2, (**c1**–**c3**) B-3, and (**d1**–**d3**) B-4 specimens.

**Figure 8:**
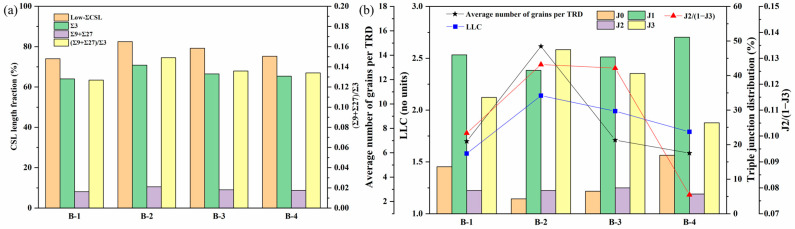
(**a**) The GBCD statistics, (**b**) average number of grains per TRD and LLC, and triple junction distribution of the TMP specimens with various annealing times.

According to the GBCD statistics in the B10 alloy treated at different rotational speeds, specimen S1400 was chosen as the object to investigate the effect of annealing time on the GBCD evolution. The IPF maps and the corresponding grain boundary reconstruction and TRD maps of specimen S1400 annealed at 750 °C for a range of time, are shown in Figure 7. Figure 8 shows the corresponding characteristics. It can be observed that the microstructure evolution depended closely on the annealing time. The average grain size without considering the annealing twins increased first and then decreased as the annealing time was prolonged. Specifically, the average grain sizes of specimen S1400 annealed at 750 °C for 30 min, 60 min, 120 min, and 180 min were 17.11 μm, 34.71 μm, 24.17 μm, and 23.42 μm, respectively. The grain size distribution also changed, going from a unimodal distribution at 30 min to a bimodal distribution for specimens annealed for 60 and 120 min and back to a unimodal distribution at 180 min (Figure 7(a1–d1)).

A change in the morphology of Σ3 boundaries can be observed in Figure 7(a2–d2). When the annealing time was 30 min, most Σ3 boundaries manifested as straight twin pairs, commonly regarded as coherent twin boundaries with limited mobility, so it is hard to improve the grain boundary interaction efficiently. An increasing fraction of Σ3 boundaries turned into curved lines as the annealing time was increased to 60 and 120 min, indicating that a higher fraction of incoherent Σ3 boundaries was formed. Some incoherent Σ3 boundaries were annihilated when the annealing time was further extended to 180 min. In terms of GBCD statistics (Figure 8a), the fraction of low-Σ CSL boundaries increased from 74.03% to 82.50% as the annealing time increased from 30 min to 60 min. Nevertheless, when increasing the annealing time to 120 and 180 min, the fraction of low-Σ CSL boundaries decreased to 79.21% and 75.25%, respectively. As the annealing time increased, the (Σ9 + Σ27)/Σ3 ratio initially increased and then gradually decreased, with the maximum value of 0.15 being attained in specimen B-2, resulting in the highest degree of fragmentation of random boundary network connectivity.

The average number of grains per TRD and LLC value has been determined for the specimen S1400 annealed at 750 °C for different annealing durations, as shown in Figure 8b. With an increase in annealing time, the average number of grains per TRD and LLC increased gradually and then decreased, i.e., specimen B-2 had the maximum number of grains per TRD and LLC value. A greater number of grains per TRD and a higher value of LLC in the specimen indicate a more significant degree of multiple twinning. Moreover, to track the evolution of random boundary network connectivity with the extension of annealing time, the triple junction distribution is also plotted in Figure 8b. A higher value of J2/(1 − J3) signifies a greater disruption in the connectivity of a random boundary network. Specimen B-2 exhibited the maximum J2/(1 − J3) value of 0.31, indicating an effective interruption of random boundary network connectivity. By comparison, specimens B-1, B-3, and B-4 showed relatively intact connectivity of a random boundary network. It is evident from the grain boundary reconstructed maps of these specimens shown in Figure 7(a2–d2). Based on the results above, it can be deduced that specimen B-2 has excellent resistance to intergranular failure.

The operation of SIBM inducing multiple twinning events during annealing treatment after FSP is identified as a critical process for optimizing GBCD by forming abundant low-Σ CSL boundaries and interrupting the connectivity of a random boundary network. For shot annealing time (30 min, specimen B-1), the low stored energy produced by FSP can hardly promote the long-distance migration of grain boundaries, leading to the formation of low-Σ CSL boundaries being limited (see Figure 8a). Extending the annealing time to 60 min (specimen B-2) allows SIBM to be activated adequately in areas with relatively high stored energy. Following this, migrated grain boundaries sweep into surrounding deformed grains until they impinge with other migrating grain boundaries. In the process of rapid grain boundary sweeping, a high fraction of low-Σ CSL boundaries are formed, and these low-Σ CSL boundaries can encounter and interact with other grain boundaries to form new low-Σ CSL boundaries. As a result, as seen in Figure 7 and Figure 8, the GBCD in specimen B-2 was further optimized to evolve into a larger number of grains per TRD and LLC value, and the connection of the random boundary network was continually disrupted, displaying an increased value of J2/(1 − J3). When annealed for 120 and 180 min, the stored energy was almost consumed, and the grain boundary migration rate became slower after full recovery, which is not conducive to forming annealing twins [43]. Meanwhile, for the reduction of the interface energy of the system, the disappearance of low-ΣCSL boundaries can occur during grain growth [38]. It can explain the slight decline in the fraction of low-Σ CSL boundaries after further extending the annealing time from 120 min to 180 min (Figure 8a).

## 4. Conclusions

In this study, the effects of the rotational speed of FSP and annealing time on the evolution of GBCD in B10 alloy was investigated. The main conclusions drawn were as follows:

(1) The GBCD of B10 alloy can be optimized effectively using FSP combined with the subsequent annealing treatment. Specifically, the highest fraction of low-ΣCSL boundaries (82.50%), mainly of the Σ3^n^ (n = 1, 2, 3) boundaries, was obtained through combining FSP at a rotational speed of 1400 rpm with annealing at 750 °C for 60 min. The connectivity of the random boundary network was effectively interrupted, which led to the optimization of GBCD. This is the consequence of prolific multiple twinning events triggered by SIBM, resulting in a large average number of grains per TRD, a high LCC value, and a great value of J2/(1 − J3).
(2) The evolution of GBCD in the B10 alloy was significantly affected by the rotational speed of FSP. With the rotational speed of FSP increased from 600 rpm to 1400 rpm, the fraction of low-ΣCSL boundaries increased monotonously. The microstructure of GBE was achieved through SIBM rather than SRX. To achieve the optimization of GBCD, it is critical to introduce suitable stored energy during TMP to avoid the occurrence of SRX phenomena while activating sufficient SIBM.
(3) Specimens processed through FSP at a rotational speed of 1400 rpm, followed by annealing at 750 °C from 30 min to 60 min, led to an increase in the fraction of low-Σ CSL boundaries, which decreased with extended annealing time up to 120 min and 180 min. Significant grain growth occurred with increasing annealing time from 30 min to 60 min.

## Figures and Tables

**Table 1:** Chemical composition of B10 alloy used in this study.

Elements	Ni	Fe	Mn	Pb	P	S	C	Sb	Cu
wt. %	9.95	1.32	0.6	0.01	0.01	0.01	0.03	0.005	Bal.

**Table 2:** Thermomechanical processing parameters for different GBE conditions.

Group	Serial Number	Rotational Speed/rpm	Annealing Temperature/°C	Annealing Time/min
A	A-1	600	750	60
	A-2	800		
	A-3	1000		
	A-4	1200		
	A-5	1400		
B	B-1	1400		30
	B-2			60
	B-3			120
	B-4			180

**Table 3:** Average grain size and length fraction of different kinds of grain boundaries for the FSPed specimens.

Specimen	Grain Size/μm	Σ1/%	Σ3/%	(Σ9 + Σ27)/%	Low-Σ CSL/%
BM	16.47	3.34	44.70	5.30	52.82
S600	1.75	30.71	8.24	0.44	12.57
S800	2.19	23.31	15.03	0.64	19.02
S1000	3.19	20.08	24.89	0.39	29.51
S1200	2.61	22.13	16.86	0.79	22.21
S1400	2.17	22.38	16.38	0.48	20.55

## Data Availability

Data are contained within the article.
